# Public health palliative care interventions that enable communities to support people who are dying and their carers: a scoping review of studies that assess person-centered outcomes

**DOI:** 10.3389/fpubh.2023.1180571

**Published:** 2023-07-26

**Authors:** Anna Peeler, Alexandra Doran, Lee Winter-Dean, Mueed Ijaz, Molly Brittain, Lorraine Hansford, Katrina Wyatt, Libby Sallnow, Richard Harding

**Affiliations:** ^1^Cicely Saunders Institute of Palliative Care, Policy, and Rehabilitation, King's College London, London, United Kingdom; ^2^GKT School of Medical Education, King's College London, London, United Kingdom; ^3^Wellcome Centre for Cultures and Environments of Health, University of Exeter, Exeter, United Kingdom; ^4^Department of Health and Community Sciences, University of Exeter Medical School, Exeter, United Kingdom; ^5^St Christopher's Hospice, London, United Kingdom; ^6^End-of-Life Care Research Group, Vrije Universiteit Brussel, Brussels, Belgium; ^7^Marie Curie Palliative Care Research Group, University College London, London, United Kingdom

**Keywords:** public health, palliative care, end-of-life, interventions, community engaged

## Abstract

**Background:**

Public health palliative care views communities as an integral part of care delivery at the end of life. This community-provider partnership approach has the potential to improve end-of-life care for people who are dying and their carers.

**Objective:**

To identify and appraise the current literature related to public health interventions that enable communities to support people who are dying and their carers.

**Methods:**

A scoping review was conducted, applying Arksey and O'Malley's methods. Data was extracted and synthesized using narrative techniques, and results are reported using PRISMA guidelines.

**Results:**

The search yielded 2,902 results. Eighteen met inclusion criteria and were included in the analysis. Interventions were categorized according to their target population: people with life-limiting illness (ex. facilitated social interaction, helplines and guided discussions about death and dying); carers (ex. social support mapping, psychoeducation, and community resource identification and facilitation); or dyads (ex. reminiscence activities, practical and emotional support from volunteers, online modules to bolster coping mechanisms). Public health palliative care approaches were delivered by key community stakeholders such as community health workers, volunteers, peer mentors, and pre-established support groups. Despite reported challenges in identifying appropriate tools to measure effectiveness, studies report improvement in quality of life, loneliness, social support, stress and self-efficacy.

**Conclusion:**

We found that community-engaged palliative care interventions can lead to appreciable changes in various outcomes, though it was difficult to determine in which contexts this approach works best because of the dearth of contextual information reported. Based on the varied design and implementation strategies, it is clear that no one method for enhancing end of life care will benefit all communities and it is crucial to engage community members at all stages of the design and implementation process. Future research should be grounded in appropriate theory, describe contextual differences in these communities, and should specifically examine how demographics, resource availability, and social capital might impact the design, implementation, and results of public health palliative care interventions.

## What is already known about this topic?

- Public health palliative care is a model of care that views communities as an integral part of care delivery at the end-of-life.
- Some people, like those in rural and coastal communities, people bound to their homes due to disability or transportation limitations, and those from historically underserved populations, might have palliative care needs that are difficult to meet with traditional services.
- Models of palliative care that include public health interventions have the potential to better serve the needs of groups that traditional services are unable to effectively meet.

## What does this paper add?

- Though varied in targeted need and approach, each included intervention improved at least one aspect of care for people at the end of life and/or and their carers, demonstrating the utility of a public health palliative care approach in different settings. Despite this, few studies discussed the possible mechanisms of action leading to improved outcomes.
- There was marked heterogeneity in the studies' theoretical underpinnings, methods and outcomes of interest which emphasizes the diversity of the public health palliative care approach and how contextual factors such as demographics, resource availability and social capital likely impact success.
- This review demonstrates the wide number of actors beyond professional services who are involved in end-of-life care, including paid community members, trained volunteers, and peer support mentors.
- Contextual data was not reported consistently among included studies thus limiting our ability to make inferences about which types of approaches work for different communities and why.

## Introduction

Palliative care is recognized by the World Health Organization as an essential health service under Universal Health Coverage (1, 2). However, globally only about 14% of people who would benefit from palliative care actually receive it (3). An estimated 56.8 million people around the world require palliative care each year, the majority of whom live in low- and middle-income countries (3). The need to correct the inequitable distribution of end-of-life care services is also evident in high-income countries, where lower socioeconomic position is associated with poorer outcomes in end-of-life care (4, 5). A more integrated approach between healthcare providers and the communities they serve is vital to bridge the gap between demand and supply (6).

Public health palliative care views communities as an integral part of care delivery at the end-of-life (7). The public health palliative care approach is informed by the five pillars for health promotion in the Ottawa Charter (8). It emphasizes the importance of health-centric public policy, supportive environments, community action, personal skill development, and health and social care services reoriented toward health promotion in order to improve the physical, mental, and social well-being of populations, including the context of life-limiting illness. This community-engaged approach has the potential to expand capacity and better serve groups such as rural and coastal communities, people living in economic poverty, people with disability or transportation limitations, and those with limited access to high-quality healthcare services (6). Furthermore, the COVID-19 pandemic shifted care into the home, accelerating new models of care delivery to meet needs beyond resource-limited health and social care institutions (9).

The level of community engagement in supporting the dying falls along a continuum ranging from information provision to consulting, co-producing, collaborating and finally empowerment (where communities lead the work) (10). The public health approach to palliative and end-of-life care ideally involves the input from members of the target community at every stage of design, implementation, and dissemination, thereby ensuring that services are relevant, people feel empowered and supported, with sustainable change. This ecological view of health acknowledges that traditional models of healthcare have limitations, and recognizes that community input is not only valuable, but essential in meeting health needs.

Previous large scale public health initiatives like the Neighborhood Network in Palliative Care in Kerala, India, and Compassionate Communities and Cities have demonstrated that a public health approach can increase the depth and breadth of a palliative and end-of-life care response for those who need it most (11, 12). Working in active partnership with key community stakeholders enables development of community driven support for people with palliative care needs within their networks and neighborhoods (13, 14). The identification and harnessing of community-specific assets with recognition of shared concerns may better reflect and serve the needs and wishes of ethnically and socially diverse populations than traditional service responses alone (10).

All along the spectrum of community engagement, public health palliative care approaches have been found to improve outcomes for both people with life-limiting illness and their carers, including improving quality of life (QoL), reducing fatigue and isolation, and increasing the size of caring networks (6). However, delivery of feasible, acceptable and effective public health palliative care programmes requires greater understanding of what might work, for whom and how, within differing social contexts, and application of robust methods to understand mechanisms and evaluate outcomes (15). This review aimed to identify and appraise the current literature related to public health interventions that enable communities to support people who are dying and their caregivers.

## Methods

### Design and research questions

A scoping review was undertaken in line with Arksey and O'Malley's methods (16); results were synthesized using narrative synthesis applying a public health palliative care framework (6). The search and screening results are reported according to the PRISMA guidelines.

The review sought to answer the following *a priori* research questions: (1) What are community needs to support those living with terminal illness? (2) What interventions have been developed and what are the theoretical models that underpin them? (3) What is the evidence for effectiveness? (4) What context-specific evidence is available for communities living with economic poverty? (5) What are the mechanisms of action? (6) What research methods are most appropriate to improve support in palliative care? (7) Which process and outcome measures are appropriate to evaluate the impact of these approaches?

The analysis was guided by Sallnow and colleagues' public health palliative care framework (6). In their 2016 systematic review (informed by the tenets of the Ottawa Charter), they explored current evidence at the cross-section of palliative care and public health, specifically seeking interventions in the Charter's community action pillar. For this review, we expanded our conceptualization of public health palliative care approaches to include the personal skill development pillar of the Ottawa Charter as well as community action, acknowledging the overlap that exists in practice. Therefore, we included interventions such as educational materials which had been created specifically for people at the end of life and/or their carers (provided that the materials had been created by or with input from community stakeholders). In their review, Sallnow and colleagues identified and described three main domains which the interventions targeted: “*Making a practical difference*, which describes the impact such work has on the immediate experiences of those facing the end of life and their carers; *Individual learning and growth*, which describes the journey of personal reflection, development and confidence that those involved in delivering the care embark on; and, *Developing community capacity* which refers to the impact of the work beyond the individuals involved, to the wider community where sustainable change can occur.” Based on this framework, we sought to assess the methods used in interventions to support the dying and their carers, understand their theoretical underpinnings, and begin to define the mechanism of action which could lead to an improvement in access to and quality of care.

### Search strategy

The search strategy is reported in Appendix 1. It was undertaken in September 2022, using the following databases: Medline, Embase, PsycInfo, Cumulative Index to Nursing and Allied Health Literature and Nursing and Allied Health Database. Additionally, we searched similar systematic reviews and publications about community engagement in palliative care for studies we might have missed in the database search. Identified publications were uploaded into Covidence and each assessed for relevance by at least two reviewers (AP, LW, AD, MI, MB). Discrepancies were discussed in weekly team meetings and resolved by consensus. Inclusion criteria were: (1) reporting the results of a public health palliative care intervention targeting the community action or personal skills domain of the Ottawa Charter, (2) the intervention aimed to directly improve the care of adults aged 18 or older at the end of life [within 1 year of dying (17)] and/or their carers, (3) outcomes focused on people at the end of life or their carers, (4) written in English language. Exclusion criteria were: (1) the intervention delivered in a healthcare setting (e.g., hospitals, hospices, skilled nursing facilities) or by healthcare professionals alone, (2) intervention was created with no community input or engagement, (3) outcomes that directly impact people at the end of life or their carers were not evaluated, (4) the intervention was aimed at bereaved individuals only, (5) the publication was a review or meta-analysis of existing literature, (6) full text was not available, or the publication was not peer reviewed (e.g., conference abstracts, gray literature). The full search and screening results are reported in a PRISMA flowchart (**Figure 2**).

### Data extraction and analysis

**Figure 1:**
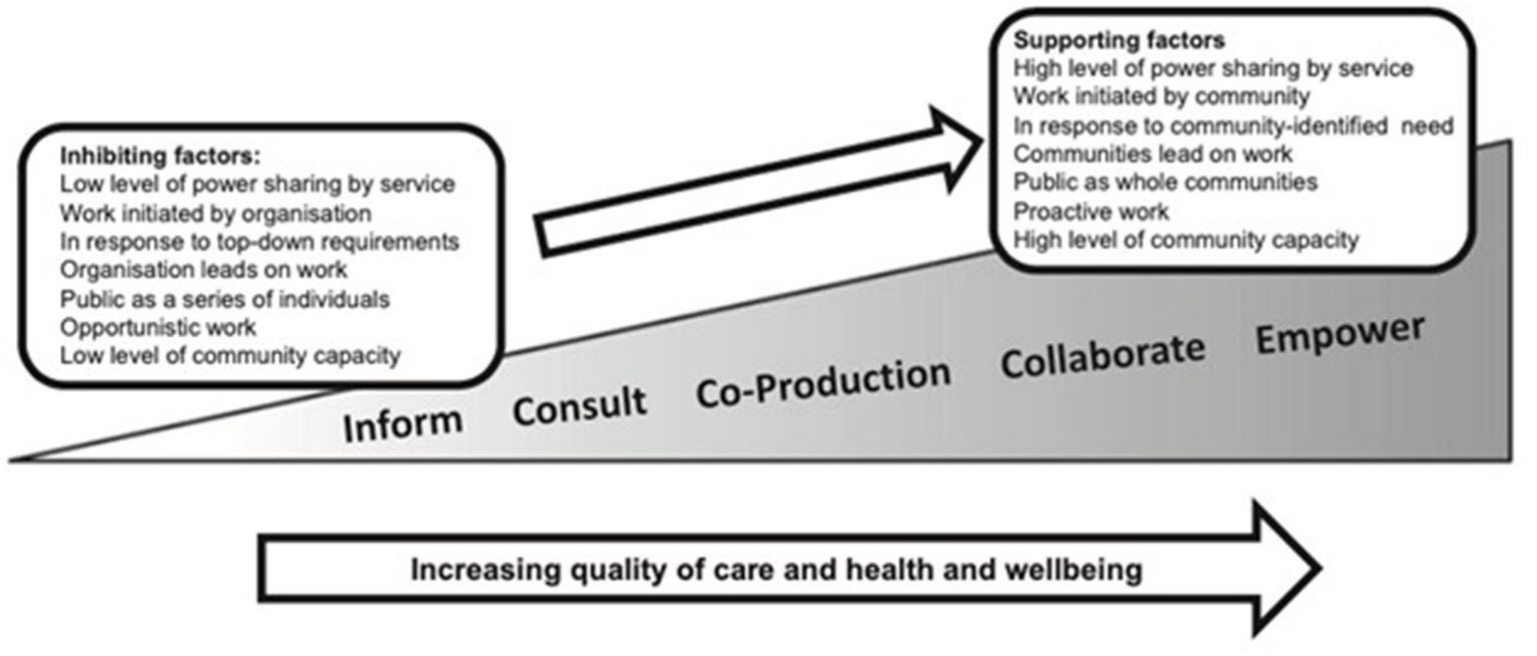
Sallnow and Paul's spectrum of engagement in end-of-life care.

In line with Arksey and O'Malley's methods (16), the study team extracted data on key aspects of the included studies' settings, designs, theoretical underpinnings, methods, outcome measures and results to address the review's research questions. We used narrative synthesis guided by Sallnow et al.'s public health palliative care framework to identify types of interventions that address the three domains of community-engaged end-of-life care (practical needs, personal growth, and community capacity), to assess the breadth of research on the topic, and to analyse similarities and differences between studies (6). Additionally, we mapped the level of community engagement involved in each of the studies based on Sallnow and Paul's spectrum of engagement in end-of-life care, seen in Figure 1 (10). We report relevant information from the selected publications according to the Preferred Reporting Items for Systematic Reviews and Meta-Analyses guidelines (18).

## Results

### Search strategy

**Figure 2:**
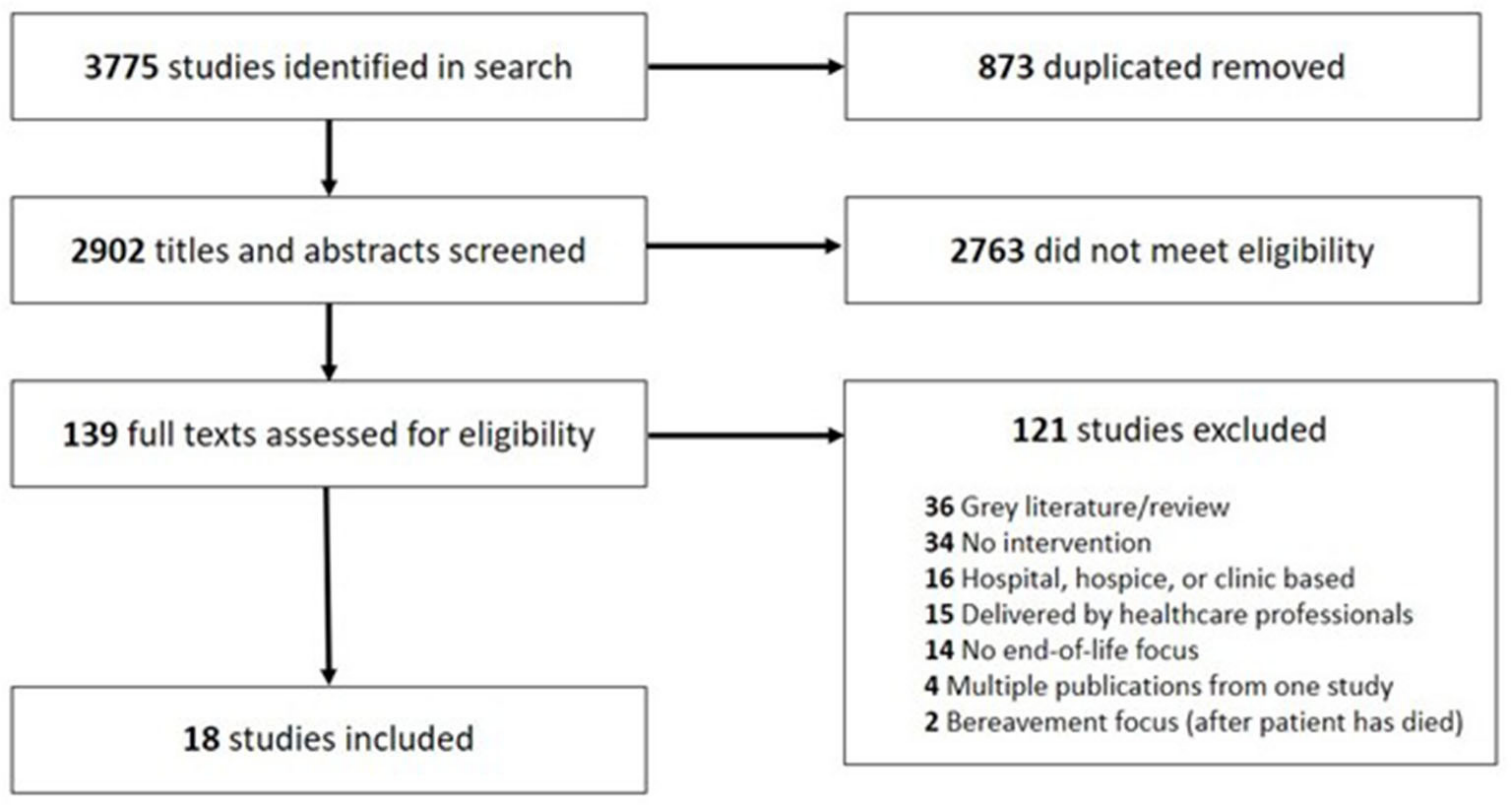
PRISMA diagram.

The search yielded 2,900 unique results, and two additional studies were identified from reference lists of other similar reviews and from expert consultation. Of those, 2,763 did not meet the eligibility criteria based on title and abstract screening. Subsequently, 139 full text articles were reviewed, and a further 121 did not meet the criteria for inclusion. In total, 18 studies were retained for analysis (see PRISMA flowchart in Figure 2).

### Study characteristics

Study summaries are presented in Table 1. Nine studies were conducted in North America, 6 in Europe, 1 in Africa, 1 in Australia and 1 in Asia. Most (*n* = 12) were conducted in large metropolitan areas focusing mainly on urban communities. The majority of these community-based interventions targeted carers of patients with life limiting illness (*n* = 10), three to people at the end-of-life only, and five to dyads. In total, 1,641 patients and 867 carers participated in these intervention studies. In seven of the studies paid community members hired by the research team delivered the intervention, in six volunteers, and in a further five no person delivered the intervention. In the studies with no specified person delivering the intervention, educational materials (i.e., informational booklet, online modules, etc.) were created based on input from community stakeholders and were freely available for community members to utilize as needed. Most of the 18 retained studies were evaluated using randomized control trials (*n* = 8), six qualitative only, two quasi-experimental, and two pre-post designs.

**Table 1:** Study details (*n* = 18).

**Year**	**First author**	**Setting**	**Study design, sample size**	**Outcomes**	**Theoretical underpinnings**	**Mechanism of action**	**Delivered by**	**Delivered to**	**Main findings**
2014	Allen (19)	USA	RCT, 45 dyads	Center for Epidemiological Studies-Depression Scale, Memorial Symptom Assessment Scale-Short Form, Brief Multidimensional Measure of Religiousness and Spirituality, Meaning in Life Scale, Caregiver Stressor Scale, Positive Aspects of Caregiving scale	Folkman's stress process model	Not discussed	Retired Senior Volunteers	Patient-carer dyads	• Patients in the intervention group had a greater reduction in emotional symptoms (*p =* 0.02) and emotional symptom bother (*p =* 0.04) and improved spiritual functioning • Caregivers in the intervention group had better Meaning of Life scale scores (*p =* 0.02)
2022	Chen (20)	China	RCT, 47 dyads	Qualitative Interviews, satisfaction questionnaire, QoL, Zarit Caregiver Burden, Family Adaptability and Cohesion Evaluation Scale II	Erikson's psychosocial development theory and Bowen's family system theory	By improving communication and bidirectional emotional support between patient and carer, encouraging gratitude, and relieving caregivers' stress, the program enabled patients to affirm positive experiences, accept or let negative experiences go, and thus help patients achieve self- integration, and perceive a better QoL	Online modules	Patient-carer dyads	• 5 themes emerged in qualitative interviews: (1) accepting and enjoying the program; (2) better communication; (3) feeling grateful for each other; (4) improved emotional support; and (5) decreased caregivers' stress • QoL (*p < * 0.001), family adaptability(*p =* 0.001), and family cohesion (*p < * 0.001) improved • Caregivers' care burden decreased in the intervention group (*p =* 0.018)
2022	Dionne-Odom (21)	USA	RCT, 46 dyads	Intervention completion rates, qualitative interviews, likeliness of recommending intervention, Rini Decision Influence Scale, Hospital Anxiety and Depression Scale	Social Support Effectiveness Theory and the Ottawa Decision Support Framework	Not discussed	Trained PC coaches	Family carers	• Carers completed 78% of intervention sessions • Carers reported a likelihood of recommending the program to others of 9.9 on a scale from 1-Not at all likely to 10-Extremely likely • Some components of the intervention showed potential benefit for effective decision support and caregiver distress
2014	DuBenske (22)	USA	RCT, 322 dyads	Demographics, Caregiver Quality of Life–Cancer Scale, Short Version Profile of Mood States, Edmonton Symptom Assessment Scale (carer-reported)	Stress and coping theoretical framework	The intervention improved carers appraisal and coping by bolstering cognitive, behavioral and practical support mechanisms. The authors state that more research is needed to understand the mechanism of action	Online modules	Patient-carer dyads	• Carers in the intervention group reported lower burden (*p =* 0.021) and negative mood (*p =* 0.006) than those in the control group • The effect on disruptiveness was not significant
2017	Grande (23)	UK	RCT, 681 people at the end of life	Novel survey with questions related to adequacy of support received by carer, physical and mental wellbeing of carer in bereavement, place of death, carers feelings regarding place of death	Not discussed	Not discussed	Volunteers	Patients	• Intervention group displayed a small but significant reduction in level of early grief and increased physical/mental wellbeing scores compared to control group • No difference in feelings of needs being met between groups
2011	Greene (24)	Australia	Quasi-experimental, 66 carers	Duke Social Support Index, Catholic Health Care Coalition Family Caregiver Questionnaire, AMA Carer Self-check, novel survey questions	Not discussed	Not discussed	Community network facilitators	Carers	• Participants in the intervention group showed improvement in caregiver fatigue, sufficient support from others, decreased resentment in the role, greater confidence in asking for assistance and were better able to find resources and support • No between-group changes were seen
2014	Hanson (25)	USA	Pre-Post, 218 people at the end of life	Novel survey with questions related to support needs and awareness of services to help with pain and symptoms, Functional Assessment of Chronic Illness Therapy–Spiritual Well-being Scale	Socioecological theory of community health promotion using existing social networks	Not discussed	Lay health advisors	Black patients	• Post-intervention 25% of patients identified hospice as source of support for pain/symptom management (pre-intervention = 4%, p = 0.04) • 60% of patients reported unmet needs for help with errands or household tasks at enrollment, while only 20% and 15% reported these areas of unmet need after 2 months in the program • QoL scores were unchanged
2011	Henricksson (26)	Sweden	Descriptive, 29 carers	Qualitative interviews	Not discussed	Not discussed	Peers	Family carers	• Participants reported that the intervention was relevant, the relationships formed were valuable, and the open approach produced a warm and relaxed atmosphere
2012	Jack (27)	Uganda	Descriptive, 21 people at the end of life	Qualitative Interviews	Not discussed	Not discussed	Community volunteers	Patients	• Participants reported that the community volunteers were very beneficial, linking them and their families to practical help, counseling and education, and hospice services when appropriate
2015	Luker (28)	UK	Quasi-experimental, 29 carers	Qualitative interviews	Not discussed	Not discussed	Informational Booklet	Carers and district nurses	• Carers were positive about the booklet, but many reported they would have liked it earlier • Carers reported feeling more positive about caregiving and more reassured and competent in their role • District nurses found the booklet useful and reported receiving fewer phone calls from study carers than others in similar situations
2022	Parker Oliver (29)	USA	RCT, 78 carers	Generalized Anxiety Disorder scale, Patient Health Questionnaire, Caregiver Quality of Life Index-Revised, and the Zarit Burden Interview	Not discussed	Not discussed	Peers	Family carers	• Participating in Facebook support groups was associated with decreased anxiety and depression carers • There was no significant difference in carer QoL or burden
2017	Pesut (30)	Canada	Pre-Post, 21 dyads	Qualitative interviews, novel survey with questions related to self-efficacy and satisfaction with the intervention, McGill Quality of Life Questionnaire	Not discussed	Not discussed	Volunteers	Patients and carers	• Carers were highly satisfied with the intervention • Carers reported that the intervention helped them with decision making, social support, engaging with life, and re-framing the experience of living with illness
2008	Ryan (31)	UK	Descriptive, 81 carers	Qualitative interviews	Not discussed	Not discussed	Lay interventionists	Carers	• Carers appreciated the emotional support, time, practical help, financial advice, and education that the program provided • Carers and health professionals both felt the program provided essential social support
2009	Steinhauser (32)	USA	RCT, 82 participants	Qualitative interviews	Byock's theory of human development and physical decline	The semi-structured nature of the sessions provided catharsis for participants by empowering them to disclose anything they felt was appropriate. The sessions allowed participants to explore their sense of self with is often interrupted at the onset of illness because crisis supersedes normal roles—by reconnecting to these roles through the personal narrative exercises, participants find emotional and spiritual growth	Lay interventionists	Patients	• Discussions of life completion may improve important health outcomes for patients at the end of life
2016	Walshe (33)	UK	RCT, 179 people at the end of life	World Health Organization QoL Brief Scale, De Jong Gierveld 6-item Loneliness Scale, Medical Outcomes Study Social Support Survey, self-reported healthcare utilization	Not discussed	Not discussed	Volunteers	Patients	• No significant differences in outcomes were found between groups at 4 weeks • Rate of change of QoL slowed in intervention group
2021	Wang (34)	USA	Descriptive, 22 carers	Qualitative interviews	Body-Mind-Spirit Model	Carers are unable to care for their loved ones if they do not first take care of themselves. The intervention targeted the bodies, minds, and spirits of carers, so they were better equipped with the self-care skills they needed to sustainably car for their loved ones	Online modules	Chinese immigrant carers	• The most beneficial aspects were self-care curriculum related to caregiving stress, lifestyle and health behavior change, community resource support, death education and end-of-life care, and spirituality and spiritual care • Caregivers appreciated the educational aspect of the intervention and wanted more assistance accessing community resources
2011	Williams (35)	Canada	Descriptive, 57 carers	Qualitative interviews	The population health promotion model (outlined in the Ottawa Charter)	When carers feel overburdened by practical concerns (financial strain, missing work, etc) they are unable to effectively care for their loved ones. This intervention helps relieve carers' burden so they have more time and energy to focus on their role in supporting their loved one without feeling burned out	Government	Carers working full time	• Carers discussed social determinants that affected their experience like gender, income and social status, working conditions, health and social services, social support network, and personal health practices and coping strategies • They rated the intervention highly and felt it benefitted them
2004	Witkowski (36)	Sweden	Descriptive, 48 carers	Qualitative interviews	Not discussed	Not discussed	Support group leaders	Carers	• Carers felt that the programme was beneficial to their own health promotion, that it was an important complement to usual palliative home care, and that they benefited from mutual experiences shared among group members

MRC, Medical Research Council; QoL, Quality of life; RCT, Randomized control trial.

In terms of reported contextual factors and demographic data, most studies (*n* = 16) reported age and sex of the participants. Nine reported marital status, six reported race and/or ethnicity, five highest level of education, four employment status, three living arrangements, two language proficiency, two religious affiliation, and one time since immigration. Two studies listed no demographic information about the participants. Four studies briefly described the catchment area for recruitment in terms of rurality and healthcare services available.

Included studies fell along the spectrum of community engagement seen in Figure 1 (10), from low levels of consultative engagement [educational materials that were created with community input then passively made available to those who might need it (20, 28)] to relatively high levels of collaborative engagement [researchers created training programs aimed at equipping volunteers with the skills they need to asses needs and then provide tailored support for people at the end of life and their carers (27, 30)]. No studies fell into the extremes of the spectrum, partly because we excluded studies that only informed participants without community engagement at any point in the intervention development.

### Interventions focused on people at the end of life

Three of the 18 included studies reported the results of interventions explicitly aimed at supporting people who were dying (25, 32, 33). All aimed to address people's practical needs by providing psychological and social support from trained community volunteers. One targeted personal growth (32) and one addressed community capacity (25). Each approach lasted between 3 weeks and 2 months and were all held in-person. Descriptions of each of the studies and their main components are reported in Table 2. None of the three studies used the same outcome measures.

**Table 2:** Intervention components.

**Year**	**First author**	**Description**	**Duration**	**Mode of delivery**	**Practical needs**			**Personal growth**		**Community capacity**	
					**Physical, psychological, spiritual**	**Education**	**Social Support**	**Knowledge, skills, and attitudes about death and dying**	**Personal reflection and confidence**	**Developing community activists**	**Embedding sustainable change**
**Interventions focused on people at the end of life (*****n** =* **3)**											
2014	Hanson	Peer-support model designed around pre-existing social networks aimed at extending practical, emotional, and spiritual support	Varied based on patients' needs, up to 2 months	In-person, via phone	Errands, household tasks, personal care, prayer, help organizing paperwork/ records/bills	Resources for help with pain relief, cancer treatment options and life-sustaining treatment	Emails, calls, cards, visits/calls with family members, help finding community resources			Support team model built on pre-existing social networks, often training natural helpers within communities	Support teams have been operating in both intervention locations >1 year after the end of grant funding
2009	Steinhauser	Semi-structured discussion about life story, regrets, heritage, and legacy with the intent of improving QoL	3 weekly sessions, 45 min each	In-person	Spiritual ideas addressed in “Forgiveness” interview e.g., “Are you at peace?”			Attitudes toward death addressed in “Forgiveness”, “Heritage and legacy” interviews	All three interviews were reflective around themes of life story, forgiveness, heritage and legacy		
2016	Walshe	Trained volunteers provided tailored support including befriending, practical support and signposting to services	4 weeks	In-person	Practical support and resource signposting		Befriending				
**Carer-focused interventions (*****n** =* **10)**											
2022	Dionne-Odom	Coaching family carer to enhance their decision support skills and how to support patient in decision-making process	1–5 weeks, sessions lasted 20–30 minutes	In-person, via phone		Decision making and communication training		Training for how to complete advance directives and POA	Communication training for how to discuss death and dying with loved ones		
2017	Grande	“Carer Support Needs Assessment Tool” which enables end-of-life needs to be identified/prioritized in partnership with the patient	Two 2-h sessions over 2 weeks	In-person	Psychological support, respite care, resource identification	Training for self-care, how to manage meds and symptoms	Training for how to ask for help from others				
2011	Greene	Community network facilitators assessed carers needs, helped mobilize their existing support networks, and connected them to available resources when needed	At least 3 monthly sessions	In-person	Psychological support, carer needs assessment, respite care	Carer role training, relaxation techniques	Training for how to ask for help from others				
2011	Henricksson	Group support program for family members of the dying with educational sessions	6 weeks	In-person		Educational offerings chosen by group members	Peer support	Group sessions focused on how to live with someone who is dying, and the practicalities of death	Group members were encouraged to share experiences and gain insight from others		Group members felt empowered to continue helping peers
2015	Luker	Informational booklet including causes of common patient symptoms, end-of-life considerations, and resources	n/a	Paper leaflet	Psychological and emotional support resources	Symptom management education		Leaflet includes information about death and bereavement			Leaflet is publicly available in perpetuity
2022	Parker Oliver	Online (via Facebook) support groups to educate and provide social support for family carers	4-weeks of content, in group until patient dies	Online		Weeks 1 and 2 provided links to educational material on Hospices and Pain	Peer support from other group members	Week 4 addressed topic of “Dying Process”, provided information and group shared perspectives	Each week included reflective practice on topic		Individuals can remain in the group as long as they wish, until their care recipient dies
2011	Williams	Government benefits scheme providing financial support and allowing full-time workers to take leave to care for dying loved one	6 weeks	n/a	Monetary aid (6 weeks of income support up to 55% of regular earnings)						
2008	Ryan	Non-clinical interventionists provided information (care options, accessing resources), emotional support, practical support (form filling, financial/benefit advice) and referral to other agencies	From diagnosis to patient death	In-person, via phone	Offered emotional support and practical (form filling, benefit advice)	Provided information on care options and accessing resources	Check in visits/calls and referral to other agencies				
2021	Wang	Carers interacted with an app containing modules related to self-care, caregiving role, exercise videos, and community resources	n/a	Online (in app)	Curriculum included exercise videos, breathing/relaxation techniques and spiritual care	Education on medication management, resource signposting			Reflection on caregivers' roles and boundaries, diet, exercise, sleep		App currently being reconfigured to address carer feedback
2004	Witkowski	Non-clinical interventionists facilitated peer support groups with carers of advanced cancer patients on topics chosen by carers	5 sessions	In-person	Discussions on psychological reaction to cancer diagnosis in patients and carers	Education on cancer prognosis and treatment options,	Discussion on “living with cancer diagnosis” in group format with coping theories				One group chose to continue meeting as a self-help group after programme completion
**Dyadic interventions (*****n** =* **5)**											
2014	Allen	Retired senior volunteers delivered a reminiscence activity intervention aimed at alleviating patient and carer distress	3 sessions	In-person	“Feelings checks” conducted in in-person meetings			“Problem solving” skills addressed in dyad manual	Dyads given reflective manual to complete. During sessions interventionalist discussed feelings evoked from the task		Some dyads expressed the intentional of continuing to work on their projects
2022	Chen	Nursing students led patients and families through experience-based interviews. Participants completed online modules asynchronously	8 sessions over 4 weeks	Online (in app)	“Mind space” module enabled patient expression of emotions.	Health education included in both control/intervention group		“Connecting You and Me” module allowed dyad discussion of their journey and expression of attitudes toward death, with facilitator	Guided reflection based on cancer experience, adulthood, childhood, adolescence and life summary		E-legacy module enabled patients to hand down wishes to others
2014	DuBenske	Dyads received a web-based lung cancer information, communication and coaching intervention	24 months	Online	CHESS website facilitated CBT principles to identify emotional distress and offered coping techniques	CHESS website provided ready and organized access to educational information, resources, news	Monitored discussion groups offering social support. Separate groups for patients, carers and bereaved carers	CHESS website facilitated one-to-one question and answer service with clinician	Aspects of CHESS encourage reflection on goals, obstacles and offer techniques to overcome		Web-based platform is a resource that can be used indefinitely
2011	Jack	Community volunteers trained in palliative care, HIV and cancer, basic nursing tasks and communication, provided tailored care to dyads, including, physical care, practical help, emotional support, and education	Not discussed	In-person, via phone	Physical care provided in management of illness, administration of medicine and cooking. Spiritual support and basic counseling offered to patient/family.	Dyads educated in areas of nutrition, hygiene, infection control and medicine concordance.	Dyad needs identified and referred to appropriate support groups	Stigma around HIV/AIDS addressed, carers trained and supported by volunteers in caring		Talks given within local communities	Programme is still ongoing
2017	Pesut	Trained volunteer navigators provided psychosocial support for dyads and helped connect them to available resources	1 year	In-person, via phone	Volunteers aided clients to identify and access services and resource	Volunteers helped with decision making via discussions of options and education, to empower clients to make their own decision regarding their care	Volunteers visited clients if admitted to hospital/care home, engaged in client hobbies and seniors group activity planning	Discussions about advance care planning and resources available	Strategies to improve client confidence in voicing healthcare related concerns such as via letter writing	Volunteers advocated for their clients at a community level to ensure they received all support available	N-CARE currently being scaled up and delivered across rural communities in Canada

meds, medications; POA, power of attorney.

Hanson et al. developed a peer support group to help meet the practical, emotional and spiritual needs of African Americans with advanced cancer based on the socioecological theory of community health promotion using existing social networks. They measured outcomes using a novel survey of support needs and awareness of services to help with symptoms and the Functional Assessment of Chronic Illness Therapy–Spiritual Well-being Scale. After 2 months people reported less need for practical, emotional, and spiritual support, were more aware of hospice care but had no change in QoL (25). Informed by Byock's theory of human development and physical decline, Steinhauser et al. performed a three-armed RCT comparing a life completion discussion intervention with previously validated relaxation exercises or control. The results form a synopsis of what participants discussed in the three sessions but do not compare the groups in the discussion (32). This was the only study to discuss possible mechanisms of action, and they postulated that their intervention allowed people at the end of life to explore their sense of self and reconnect with their roles outside of illness, thus facilitating personal and spiritual growth. Walshe et al.'s study randomized 196 adults with a terminal diagnosis to receive a volunteer support intervention immediately or after a 4 week wait. They collected the World Health Organization QoL Brief Scale, De Jong Gierveld 6-item Loneliness Scale, Medical Outcomes Study Social Support Survey, and self-reported healthcare utilization from participants. While the intervention produced a positive shift in QoL, loneliness, and perceived social support scores these were not statistically significant (33).

To summarize, two of the three studies used quantitative methods to assess outcomes for people at the end of life [one used a novel survey (25) while the other used validated tools (33)]. One showed improvement in people's needs being met post-intervention (25), but neither showed a statistically significant increase in QoL. One study used qualitative interviews to determine patient satisfaction with the intervention (32). Patients in this study found the interviews to be beneficial in reflecting on their lives and accepting death which the authors stated may have improved their QoL.

### Carer-facing interventions

Ten studies focused solely on carers' needs as the person they cared for approached the end-of-life (21, 23, 24, 26, 28, 29, 31, 34–36). Four approaches connected individual carers with trained community members employed by the research team (21, 23, 24, 31) while three joined multiple carers together to form small support groups (26, 29, 36). Three did not utilize interventionists and instead provided standardized support (online modules, information booklet, and government provided financial assistance) aimed at improving carers QoL and self-efficacy (28, 34, 35). All standardized support approaches were informed by community stakeholders and were freely available to community members to use as needed. Interventions lasted between one and 12 weeks and were implemented in a variety of formats, including in-person, online, and hybrid. To assess carer outcomes six studies used qualitative interviews (26, 28, 31, 34–36), two used quantitative methods [two validated tools (24, 29) and one a novel survey (23)], and one used mixed-methods (21). The studies that used validated tools chose the Rini Decision Influence Scale, Hospital Anxiety and Depression Scale, Duke Social Support Index, Catholic Health Care Coalition Family Caregiver Questionnaire, American Medical Association Carer Self-check.

Three of the 10 described the theories that underpinned them, including the Social Support Effectiveness Theory and the Ottawa Decision Support Framework (21), the Body-Mind-Spirit Model (34), and the population health promotion model (35). Two studies discussed possible mechanisms of action leading to improved outcomes. One cited improving physical and emotional well-being of carers allowing them to be better equipped to sustainably care for their loved one (34), and the other postulated that their intervention relieved the burden of practical concerns (financial strain, missing work, etc.,) thereby allowing more time and emotional energy for caring (35, 37). None of the studies that did not state a theoretical framework posited a mechanism of action.

All 10 interventions addressed at least one aspect of carers' practical needs, from psychological support and education about how to care for someone seriously ill or dying, to financial assistance. Six highlighted the importance of personal growth and encouraged participants to reflect on their experience and their own perceptions of death and dying (21, 23, 24, 26, 28, 29, 34). Five involved community capacity building, often in the form of peer support groups that could continue to meet after the person had died (26, 28, 29, 34, 36). None specifically focused on training community volunteers or activists to continue the program after the intervention study was complete.

As seen in Table 1, there was little overlap in the measures used to assess outcomes, so opportunities for comparison are limited. In all seven of the studies that employed qualitative interviews, carers were satisfied with the intervention and felt it substantially benefitted them. In the studies that used quantitative methods, researchers found that carers were likely to recommend the intervention (21), felt improved mental and physical well-being (21, 23, 29), had decreased anxiety and depression (21, 29), and decreased early grief after the patient had died (23).

### Dyadic interventions

Five of the 18 included studies focused on patient-carer dyads (19, 20, 22, 27, 30). The dyadic interventions reported the highest level of community engagement in the development of the intervention and were the most comprehensive in addressing all three domains of the guiding framework. All five touched on at least one aspect within each of the three domains. All paid specific attention to the psychological needs of people at the end of life and their carers, aimed to improve knowledge about and acceptance of dying, and made efforts to embed sustainable change into community networks by bolstering volunteers and resources available to people facing the end of life and their carers. The duration range for dyadic interventions was wide, between 3 weeks and 1 year, and they were delivered in-person, over the phone, online or through apps designed with community input or moderated by community members.

Three of the five stated the theoretical framework that informed the study, including Folkman's stress process model (19), Erikson's psychosocial development theory and Bowen's family system theory (20), and the Stress and coping theoretical framework (22). Two discussed possible mechanisms of action (20, 22). One cited communication and bidirectional emotional support improvements as the key components of improved QoL (20), while the other asserted that the intervention helped improve carers' cognitive, behavioral and practical support mechanisms, thus improving their ability to cope with stressors (22).

Two of the studies evaluated outcomes using quantitative measures only (19, 22), two using mixed methods (20, 30), and one using qualitative methods only (27). There was almost no overlap in outcome measures. Those that used validated measures chose the Center for Epidemiological Studies-Depression Scale, the Memorial Symptom Assessment Scale-Short Form, the Brief Multidimensional Measure of Religiousness and Spirituality, the Meaning in Life Scale, the Caregiver Stressor Scale, the Positive Aspects of Caregiving scale, the Zarit Caregiver Burden scale, the Family Adaptability and Cohesion Evaluation Scale II, the Caregiver Quality of Life–Cancer Scale, the Short Version Profile of Mood States, the Edmonton Symptom Assessment Scale, and the McGill Quality of Life Questionnaire. All four studies using quantitative measures reported statistically significant positive outcomes for both patients and carers. Allen and colleagues reported decreased emotional symptoms and increased spiritual functioning (19). Chen and colleagues reported high satisfaction from both people at the end of life and carers and increased QoL and family cohesion (20). DuBenske and colleagues reported lower caregiving burden and negative mood in the intervention group compared with the control group (22). Finally, Pesut and colleagues reported increased confidence in decision making and perception of social support (30). The studies using qualitative methods similarly all reported positive experiences with the intervention and experiential reports of improved communication, psychosocial functioning and acceptance of death and dying (20, 27, 30).

## Discussion

The purpose of this scoping review was to identify and appraise the current literature on public health palliative care interventions aimed at engaging community members in supporting people at the end of life and their carers. The interventions, their theoretical underpinnings, mechanisms of action, measurement strategies and results were heterogeneous. This emphasizes the wide range of interventions that comprise the public health palliative care approach and the importance of co-creating end-of-life care delivery strategies through community action and engagement to fit different contexts. Importantly, most successfully improved at least one aspect of care for people with life limiting illness and their carers, demonstrating the utility of this philosophy of care. This is supportive of the broader literature, which acknowledges the positive impact of engaging communities to improve health and well-being (6, 11, 38).

In terms of supporting people at the end of life and their carers, communities have wide ranging needs that are dependent on context, demographics, and social capital. These needs can be divided into the three distinct categories outlined in the guiding framework: practical needs, personal growth and community capacity (6). Support for practical needs, such as social interaction, symptom management, shopping and financial subsidies, were employed in all studies. For example, most included interventions involved social interaction with trained volunteers or peers in a similar situation and reported benefits for both people at the end of life and their carers. Six studies directed resources to bolstering social networks, while six provided participants with opportunities to interact with community members trained and paid by the research team and volunteers to discuss their needs and challenges. Older adults cite maintaining social well-being as more important to their overall health than their physical and cognitive state (39), however social support is often neglected in the care of older adults with debilitating conditions, leading to social isolation, decreased quality of life, and poorer health outcomes (40). Health professionals often do not have the skills or resources to support the social needs of people at the end of life and carers, nor are they the most appropriate people to do so. Building and supporting sustainable, community-based networks are a more appropriate response to these needs.

Personal growth needs, including education, training, and reflection to strengthen knowledge, skills and confidence around to death and dying, were identified and addressed in 12 of the 18 included studies. These needs were addressed with a range of strategies aimed at improving self-efficacy, such as reflection exercises, decision support coaching, and educational offerings about how to live with and support those with a terminal diagnosis. Death, dying, and loss are universal phenomena, yet strategies and resources to support people to learn and grow as they experience them, and to share this learning within their community networks, are often lacking (41). For both people at the end of life and carers, sharing these interpersonal reflections and experiences with others may provide alternative perspectives and, as found in this review, improved emotional functioning (19) and lower burden (22). Noonan et al. define the outcome of this accumulation of knowledge and skills that carers often develop through practice as ‘death literacy', and suggest that death literacy is a resource that strengthens the capacity of individuals and communities for future caring (42).

Lastly, opportunities to build community capacity, including the development of community activist networks, increasing social capital, and partnerships with professional institutions to embed sustainable change, represent an important facet to address community needs in end-of-life care. Eleven of the 18 included studies identified and addressed community capacity building as a need, most often in the form of developing a resource (i.e., educational modules, support groups, volunteer training programme) to be used by the community in perpetuity. Expanding community capacity to support equitable and sustainable end-of-life care is crucial in developing interventions that can continue even after the resources and workforce research teams offer are removed (43). The varied needs addressed in each of the studies supports the idea that public health palliative care approaches work best when they are locally generated in response to community needs and available resources (10).

Other than some similarities in the identified community needs, we found heterogeneity in almost all aspects of the design, implementation, and evaluation of the included intervention studies. Half (*n* = 8) of the publications reported a theoretical framework underpinning the approach taken, though none overlapped. Similarly, authors described differing mechanisms of action leading to improved outcomes in the five studies that reported on it. Interestingly, among the 10 studies that did not describe their theoretical underpinnings, none went on to discuss the mechanisms of action precipitating their outcomes, suggesting that grounding an intervention in a theoretical framework provided critical scaffolding to establish and test the mechanisms of action that are intended to lead to improved outcomes.

Additionally, studies fell all along the spectrum of community engagement (10) but were concentrated in the middle. In 13 of the 18 included studies, community members acted as agents to deliver the intervention, seven as paid members of the research team, and six as unpaid volunteers. Most studies demonstrated progress in consulting community stakeholders in the development of their intervention and co-creating solutions to identified problems, but nearly all fell short of collaborating with communities to promote shared-decision making in the development of new models of care to meet the needs of people at the end-of-life and their carers. None of the studies were considered to fall into the empowerment level of community engagement. While it is possible some aspects of community engagement were not detailed or that continuing efforts have not been reported, this has implications for the sustainability of the approaches. Particularly in burgeoning fields in which theoretical underpinnings are still being established and tested, as with public health palliative care, community engagement is vitally important for defining key concepts, community assets and needs, appropriate outcomes and sustainable solutions (44). Future research should focus on expanding sustainable community engagement approaches in order to more fully empower communities to identify needs and develop strategies to support people at the end of life.

Once needs were identified and interventions to address them were developed, research teams were tasked with developing evaluation strategies that examined key outcomes for people at the end of life and their carers while also acknowledging the importance of individual experiences and contextual nuance. Again, there was wide variability in the chosen outcome measurements which makes it difficult to conclude which methods are best in evaluating intervention efficacy in which contexts. Qualitative interviews were utilized in 14 studies and were the most common data collection method in evaluating the results and efficacy of the included interventions. Six studies used novel, unvalidated quantitative surveys aimed at eliciting satisfaction with the intervention or quality of care. Furthermore, among those that used validated, quantitative measures, few studies used common tools. QoL was measured using seven different tools across seven different studies (ex. Caregiver Quality of Life–Cancer Scale, McGill Quality of Life Questionnaire, World Health Organization Quality of Life Brief Scale), while caregiver burden was measured using four different tools across three different studies (ex. Caregiver Stressor Scale, Zarit Caregiver Burden, American Medical Association Carer Self-check). Many authors cited the lack of validated quantitative outcome measurement tools as a limitation.

The varied outcome measurement strategies represented in the field of included studies may reflect the differing priorities of the communities in which they were implemented. Defining outcomes that are meaningful to community members is as important as co-creating palliative care approaches that fit the community's needs. It is imperative that future research includes systematic evaluation of which measures are meaningful to community members and the co-creation of research strategies with community members to better fit their context. To do this, the WHO recommends beginning community-engaged health services development with exploratory data collection such as qualitative interviews, co-design workshops, or field observation designed to understand context (i.e., community infrastructure, social networks, existing services), encourage stakeholder engagement and buy-in, and identify shared goals and their corresponding outcome metrics (45).

The contextual information, including personal and environmental factors, that was reported varied widely between studies. In terms of personal factors, nearly all studies (*n* = 16) reported the age and sex of their participants, but other variables such as marital status, race and ethnicity, education level and employment status were collected less consistently. Environmental factors such as location, urbanicity, access to healthcare services, local infrastructure and transportation were rarely reported. Both personal factors (such as age, sex, race, ethnicity, socioeconomic status, education level, disease type or severity, political and religious ideations) and environmental factors (such as access to healthcare services and urbanicity) have been shown to affect engagement with end-of-life services and outcomes (5). As such, these variables have important implications for how interventions are developed and how their results should be interpreted. While an aim of this review was to examine who public health palliative care approaches work for, it was difficult to do so without detailed descriptions of each of the target communities. Understanding the demographic and socioeconomic context of a community is vitally important to designing appropriate end-of-life services that meet their needs. As the field of public health palliative care moves forward, researchers have an imperative to pay particular attention to these social and structural determinants, the context in which they are working, report personal and environmental variables that could impact uptake and examine associations with outcomes if possible. In doing so, we can gain a better understanding of who these approaches work for and in which contexts and move to tackle the significant inequity which exists currently.

As a matter of special interest, we hoped to focus attention on studies that specifically targeted rural and coastal communities or communities living in economic poverty. Rurality and economic status have been shown to impact access to and the delivery of in end-of-life care (4, 5). In this review, very few studies provided sufficient contextual information to determine whether or not their samples included people from these groups, and even fewer included related aims. The majority of studies took place in high-income countries (*n* = 16), and only a few reported any data related to the economic status of their participants such as highest level of education (*n* = 5) and employment status (*n* = 4). The only publication that had an in-depth discussion of economic status and its impact on the intervention was Jack and colleagues' 2011 report of community volunteers in two urban centers in Uganda (27). Here, they discussed the financial strain of the Ugandan population and the national health system and how that impacted the sustainability of the project. While public health and community-engaged palliative care can benefit people with life-limiting illness in all settings, this model of palliative care notably could benefit those living in rural and coastal communities and those in economic poverty who are less likely to receive appropriate palliative and end-of-life care (46). Included studies provided very little specific detail as to the relative economic status or rurality of the subject populations which make it difficult to determine which interventions and models of care would be most beneficial to this population.

## Limitations

First, as the aim of this review was to identify publications that report the results of interventions aimed at fostering community engagement in end-of-life care, we only included studies that reported outcomes from people at the end-of-life or their carers. There are multiple public health palliative care initiatives, like the Neighborhood Network in Palliative Care in India and Compassionate Communities in Canada and Australia, that report community level or volunteer outcomes which fell out of the scope of this review (11, 37, 47). Secondly, we excluded gray literature, conference posters or abstracts without full-text publications, and non-peer reviewed literature. Lastly, there is potentially evidence published in languages other than English that we were unable to identify.

## Conclusion

In this review, we aimed to gain a better understanding of existing public health palliative care approaches which captured individual outcomes to understand for whom and how they work, and how differing contexts might impact their design and delivery. We found that community-engaged palliative care interventions represent a strong opportunity for improving support at the end of life, and that engaging community members at various stages in the process can lead to appreciable changes in outcomes. There was marked heterogeneity in the studies' theoretical underpinnings, methods, outcomes of interest and results which suggests that this field is developing rapidly. Based on the varied design and implementation strategies and their collective success in improving outcomes, it is likely that different community engagement approaches will support public health palliative care approaches in different communities. Context is a crucial component in understanding community needs and how they might benefit from public health interventions. Future research should define contextual difference in these communities and should specifically examine how demographics, resource availability, and social capital might impact the design, implementation, and results of public health palliative care interventions. Defining these contextual differences and their impact, then adjusting community engagement strategies appropriately, public health approaches can better fit the needs of the communities in which they are situated.

## Data availability statement

The original contributions presented in the study are included in the article/Supplementary material, further inquiries can be directed to the corresponding author.

## Author contributions

The review was designed and developed by LW-D, LH, KW, LS, and RH. The search was carried out by LW-D. AP, AD, LW-D, MI, and MB reviewed all items identified in the search and systematically decided which should be included in the final manuscript. All authors contributed to the writing of the manuscript and provided substantial edits to the final draft.
