# Supplementary material for: Public health palliative care interventions that enable communities to support people who are dying and their carers: a scoping review of studies that assess person-centered outcomes
Source: Front Public Health. 2023 Jul 26;11:1180571. doi: 10.3389/fpubh.2023.1180571 (PMC10410270; doi:10.3389/fpubh.2023.1180571)
Supplement: Supplementary file 1 [file Table_1.pdf]

Appendix 1.

Search Strategy Medline search terms:

1. exp terminal care/

2. exp terminally ill/ 3. (terminal* adj

3 (care or caring)).mp.

4. last year of life.mp.

5. LYOL.mp.

6. (end of life or end-of-life).mp.

7. (hospice or hospice care).mp.

8. Palliat*

9. Dying

10. 1 or 2 or 3 or 4 or 5 or 6 or 7 or 8 or 9

11. Public health

12. Mobilise

13. Community resource

14. Community network

15. Social network

16. Health promotion

17. Social change

18. Social support

19. Community development

20. Community participation

21. Community engagement

22. Compassionate community

23. 11 or 12 or 13 or 14 or 15 or 16 or 17 or 18 or 19 or 20 or 21 or 22

24. 10 AND 23
